# Tumor cytotoxicity and immunogenicity of a novel V-jet neon plasma source compared to the kINPen

**DOI:** 10.1038/s41598-020-80512-w

**Published:** 2021-01-08

**Authors:** Lea Miebach, Eric Freund, Stefan Horn, Felix Niessner, Sanjeev Kumar Sagwal, Thomas von Woedtke, Steffen Emmert, Klaus-Dieter Weltmann, Ramona Clemen, Anke Schmidt, Torsten Gerling, Sander Bekeschus

**Affiliations:** 1 ZIK plasmatis, Leibniz Institute for Plasma Science and Technology (INP), Felix-Hausdorff-Str. 2, 17489 Greifswald, Germany; 2 Department of General, Visceral, Thoracic, and Vascular Surgery, Greifswald University Medical Center, Ferdinand-Sauerbruch-Str., 17475 Greifswald, Germany; 3 Institute for Hygiene and Environmental Medicine, Greifswald University Medical Center, Ferdinand-Sauerbruch-Str., 17475 Greifswald, Germany; 4 Clinic for Dermatology and Venereology, Rostock University Medical Center, Strempelstr. 13, 18057 Rostock, Germany

**Keywords:** Chemical physics, Plasma physics

## Abstract

Recent research indicated the potential of cold physical plasma in cancer therapy. The plethora of plasma-derived reactive oxygen and nitrogen species (ROS/RNS) mediate diverse antitumor effects after eliciting oxidative stress in cancer cells. We aimed at exploiting this principle using a newly designed dual-jet neon plasma source (^V^jet) to treat colorectal cancer cells. A treatment time-dependent ROS/RNS generation induced oxidation, growth retardation, and cell death within 3D tumor spheroids were found. In TUM-CAM, a semi in vivo model, the ^V^jet markedly reduced vascularized tumors' growth, but an increase of tumor cell immunogenicity or uptake by dendritic cells was not observed. By comparison, the argon-driven single jet kINPen, known to mediate anticancer effects in vitro, in vivo, and in patients, generated less ROS/RNS and terminal cell death in spheroids. In the TUM-CAM model, however, the kINPen was equivalently effective and induced a stronger expression of immunogenic cancer cell death (ICD) markers, leading to increased phagocytosis of kINPen but not ^V^jet plasma-treated tumor cells by dendritic cells. Moreover, the ^V^jet was characterized according to the requirements of the DIN-SPEC 91315. Our results highlight the plasma device-specific action on cancer cells for evaluating optimal discharges for plasma cancer treatment.

## Introduction

With several million deaths in 2018, cancer is the second leading cause of death worldwide^1^. In recent years, oncological research has significantly improved our knowledge of the pathogenesis in many types of cancer, resulting in various treatment strategies. However, despite the continuous advance in medicine, cancer burden continues to grow globally, and the treatment of many tumor entities remains challenging in clinical oncology. Patients and their attending physicians face difficulties like severe side effects, chemoresistance, and lack of therapeutic efficiency among existing treatment regimens. Besides other approaches, targeting the cellular redox state in tumor cells has shown promising results^2^. Reactive oxygen species (ROS) act as biological signaling molecules that influence cellular metabolism, phenotype, and proliferation^3–5^. An increase in intracellular ROS levels has also been shown to induce cell cycle arrest and apoptosis^6,7^ and immunogenic cancer cell death (ICD). Upon undergoing ICD, cells can be characterized by increased surface expression or release of so-called damage-associated molecular patterns (DAMPs) that serve as 'danger signals' for the immune system^6^. A variety of DAMPs have been shown to contribute to the immunogenicity of cell death, including calreticulin (CRT), adenosine triphosphate (ATP), and chaperons of the heat shock protein (HSP) family that can recruit immune cells to the tumor site and subsequently elicit T-cell based antitumor immunity^7–12^. Several chemotherapeutic agents, including Doxorubicin and Mitomycin C, and physical therapies like photodynamic and radiotherapy, mediate their therapeutic efficiency partially through ROS production^13–16^.

A way of directly generating exogenously applied ROS is cold physical plasma. Plasmas are produced by energizing gases resulting in their partial ionization^17^. Recent studies showed tumor-toxic effects of cold physical plasma on numerous tumor entities in vitro^18–22^ and in vivo^23–25^. Beneficial antitumor effects were also shown in the palliation of head and neck cancer patients^26–28^. In those studies, the argon plasma jet kINPen was used, which is accredited as a medical device in Europe and licensed to treat infected wounds and skin diseases up to now^29,30^. It is highly standardized and investigated for genotoxic and mutagenic safety^31–33^. The different applications in the field of plasma medicine are a consequence of ROS's hormetic action^34^. Plasma has ROS-mediated stimulatory effects in eukaryotic cells at shorter treatment times or lower energy deposition (oxidative eustress), while higher intensities or longer treatment times lead to oxidative distress and cell death^35^. Along those lines, we here investigated a novel neon-driven plasma jet that generates two instead of one plasma effluents merging at their tips in the form of a V (^V^jet). The hypothesis was that such a geometry might shorten the treatment times needed to achieve a tumor-toxic effect. Our previously used three-dimensional (3D) model systems such as 3D multicellular tumor spheroids^36^ and complex tumors grown on the chorion-allantois-membrane (CAM) of chicken embryos (TUM-CAM)^33^ in combination with sophisticated imaging and multicolor flow cytometry were used to investigate colorectal cancer. For the first time in plasma cancer treatment, not only a prototype plasma source (novel ^V^jet) was investigated but directly compared with the kINPen as a reference jet, aiming at finding a superior action of either system.

## Results

### Quantification of reactive species

**Figure 1:**
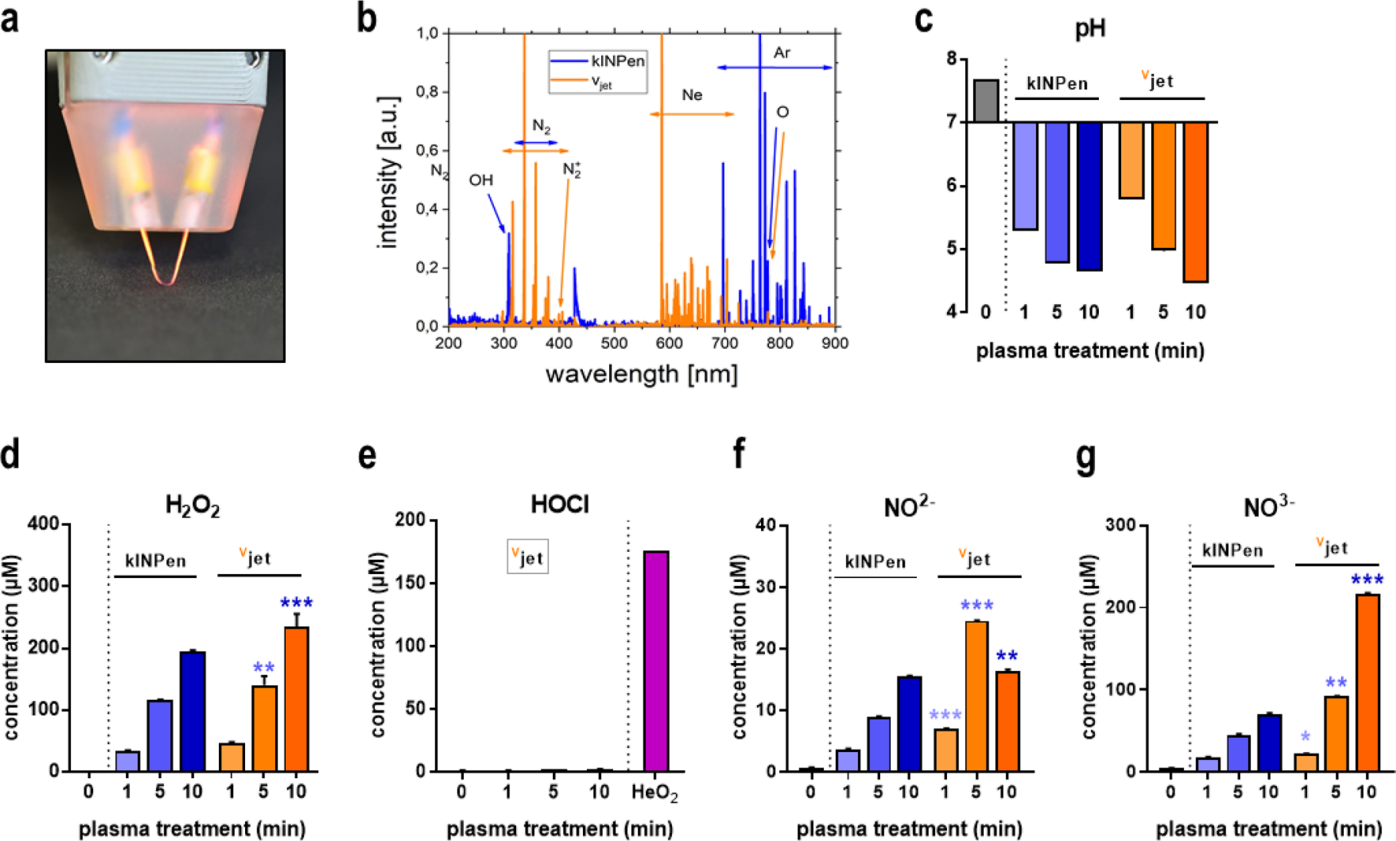
Plasma-derived ROS/RNS. (**a**) photograph of the neon ^V^jet plasma used in this study; (**b**) OES of ^V^jet (magenta) and kINPen (blue) plasma effluent normalized (1.0) to the maximum peak of each of the spectrum; (**c**) pH in ddH_2_O after 1 min, 5 min and 10 min plasma treatment; (**d**–**g**) quantification of H_2_O_2_ (**d**), HOCl (**e**), NO_2_^−^ (**f**), and NO_3_^−^ (**g**) in plasma-treated ddH_2_O. Plasma treatment of ddH_2_O using He/O_2_ as feed gas served as a positive control for HOCl. Data are representative (**b**, **c**) or mean ± standard error (SEM) from three independent experiments. Statistical analysis was performed using t-test comparing both jets at equivalent treatment times (*p < 0.05; **p < 0.01; ***p < 0.001); n.s. = non-significant.

Optical emission spectroscopy (OES) was performed to characterize the ROS chemistry derived from newly engineered ^V^jet (Fig. 1a) and kINPen plasma, and each of the spectra was normalized to the maximum peak to allow comparison on a similar scale. Recorded emission spectra between 200 and 900 nm (Fig. 1b) showed spectral lines at 300–310 nm, which indicate the presence of hydroxyl radicals (OH•). The spectral lines at 330–380 nm, mainly observed in ^V^jet plasma, correspond to the second positive nitrogen system, responsible for generating reactive nitrogen species (RNS). Spectral lines above 500 nm represent the ionized neon (magenta) and argon (blue) molecules. Some of the peaks, albeit at low intensity, represent other ROS types, e.g., atomic oxygen at 777 nm. Since target cells are surrounded by liquids such as cell culture media or interstitial fluid during in vitro or in vivo plasma treatments, physical plasma effects are mediated through ROS generated in the plasma gas phase and plasma liquid interphase, and subsequently submerge into the bulk liquid^37^. Four milliliters of double-distilled water (ddH_2_O) were plasma-treated for 1 min, 5 min, and 10 min. Plasma treatment caused acidification of liquids without significant differences between both jets (Fig. 1c). Formation of hydrogen peroxide (H_2_O_2_), mainly a secondary product of, e.g., HO•^38^, is typical in plasma-treated solutions and was observed in liquids exposed to both types of jets (Fig. 1d). Significant differences between kINPen and ^V^jet were found for long treatment times of 5 min and 10 min. By contrast, the generation of hypochlorous acid (HOCl) was not detected, neither with the Ar-driven kINPen nor with the Ne-driven ^V^jet but only with the He/O_2_-driven kINPen as a positive control (Fig. 1e). Regarding RNS-derived secondary reaction products, the plasma jets differed notably in their nitrite (NO_2_^-^, Fig. 1f) and nitrate (NO_3_^-^, Fig. 1g) production, with the ^V^jet generating higher concentrations at long exposure times.

### Cytotoxicity in 3D tumor spheroids following plasma treatment

**Figure 2:**
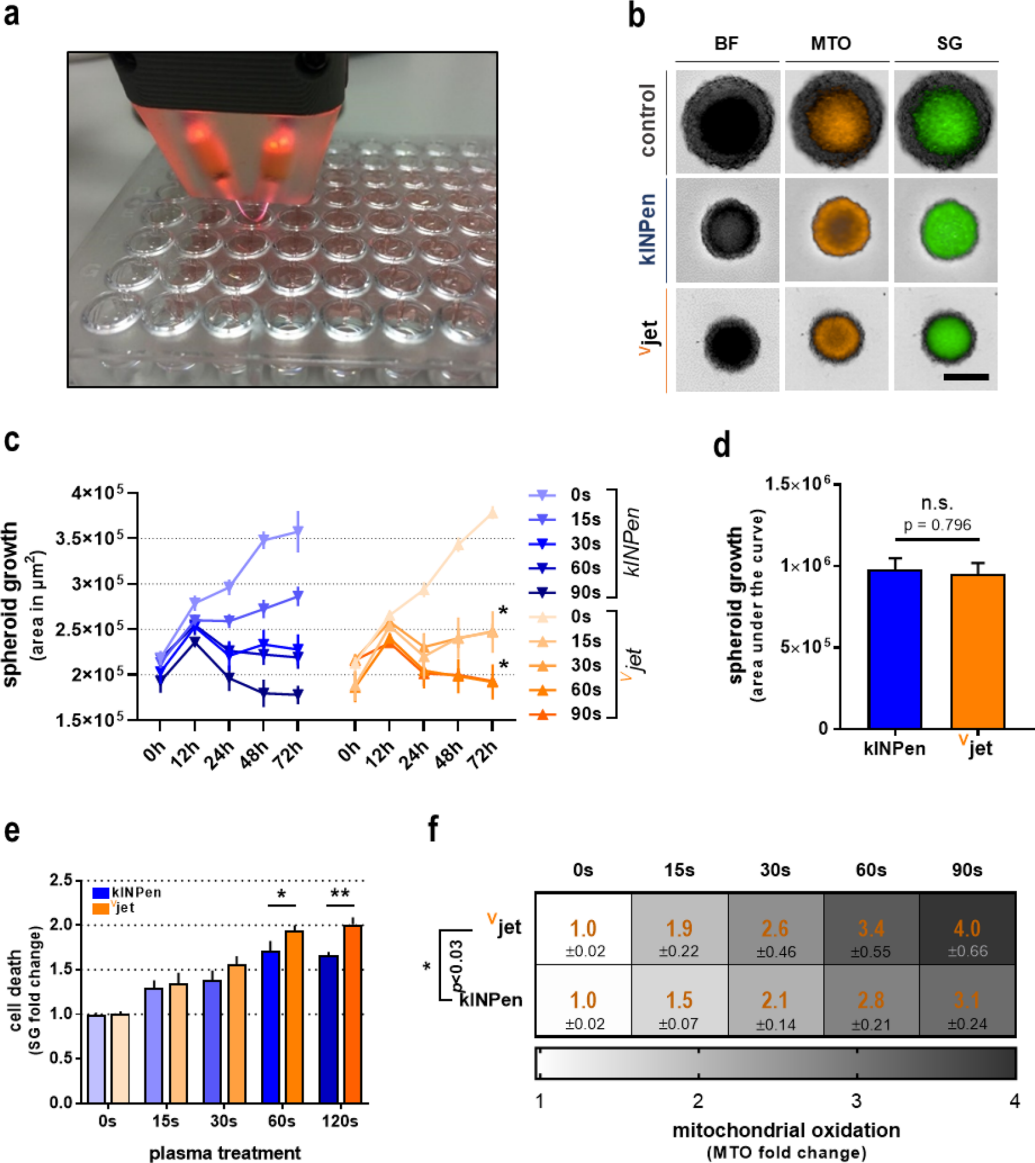
Cytotoxicity in 3D tumor spheroids following plasma treatment. (**a**) Image of the ^V^jet treatment of 3D multicellular tumor spheroids derived from CT26 colon carcinoma cells; (**b**) representative images of tumor spheroids in brightfield (BF), mitotracker orange (MTO), and sytox green (SG) fluorescence channel 24 h after plasma treatment; (**c**) spheroid growth over 72 h after plasma treatment; (**d**) cell death and (**e**) mitochondrial oxidation 24 h after plasma treatment. Data are representative of three independent experiments. Statistical analysis was performed using two-way Anova or *t-*test (*p < 0.05); **p < 0.01); n.s. = non-significant. scale bar = 200 µM.

Dependent on the plasma treatment intensity or time, respectively, plasma-derived ROS and RNS are known to stimulate or diminish cellular proliferation. To compare biological effects between kINPen argon plasma and ^V^jet neon plasma, we investigated alteration in three-dimensional tumor spheroids of CT26 colorectal cancer cells (Fig. 2a). Exposure to the kINPen or ^V^jet plasma for 15 s, 30 s, 60 s, and 90 s was followed by high content imaging (Fig. 2b). When imaged consecutively over 72 h, both kINPen and ^V^jet treatment reduced spheroid growth compared to controls (Fig. 2c). ^V^jet treatment of 15 s and 60 s showed a significantly stronger impact on growth reduction than equal exposure to kINPen plasma after 72 h. However, growth reduction over time did not differ significantly between kINPen or ^V^jet plasma exposure (Fig. 2d). To evaluate whether spheroid growth was a consequence of reduced cellular proliferation (total spheroid size) and cytotoxicity, spheroids were stained with the cell death discrimination dye sytox green (SG). Increased cell death in plasma-treated spheroids was observed (Fig. 2e). Again, ^V^jet plasma treatment was significantly more cytotoxic regarding prolonged exposure for 60 s and 90 s than equal treatment duration with kINPen plasma. As it is commonly known that the abundance of ROS produced by physical plasma exerts its effects through the induction of oxidative distress, we also investigated mitochondrial oxidation in tumor spheroids after plasma treatment. A substantial increase in mitochondrial oxidation was observed in each treatment regime (Fig. 2f). Supporting the findings in cytotoxicity analysis, the ^V^jet plasma treatment showed significantly higher intra-tumoral oxidation than the kINPen.

Taken together, we confirmed the substantial effects of plasma treatment on tumor cell proliferation, cell death, and the cellular redox state. A comparison of respective treatments indicated a modestly enhanced cytotoxic capacity of ^V^jet neon plasma over the argon plasma jet kINPen.

### Cancer cells undergo immunogenic cell death in response to plasma treatment

**Figure 3:**
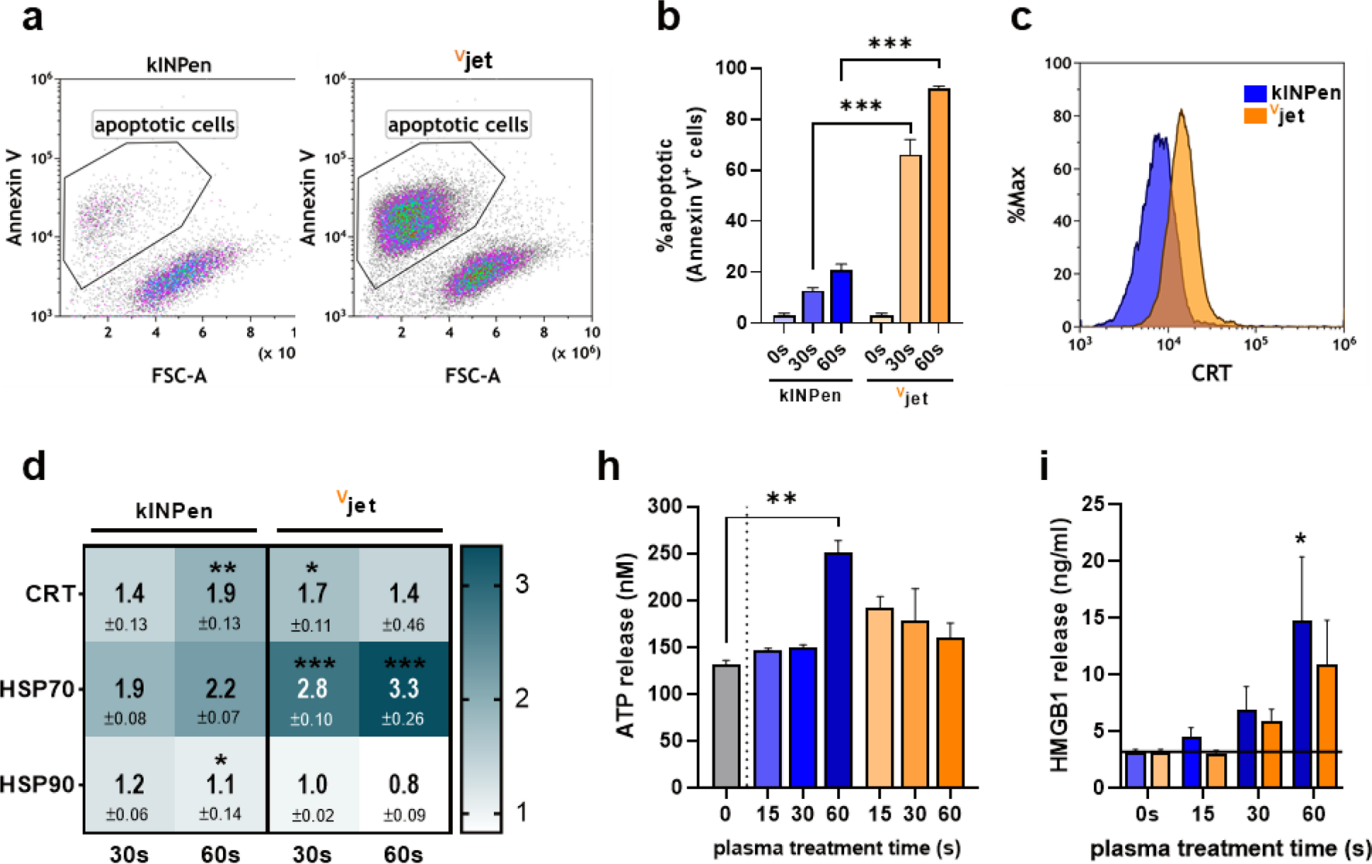
Immunogenic cell death in cancer cells following plasma treatment. (**a**) representative flow cytometry dot plots of annexin V + cells 24 h after plasma treatment and (**b**) quantification thereof; (**c**) representative flow cytometry intensity histogram of CRT surface expression and (**d**) quantification of ecto-CRT, HSP70, and HSP90 normalized to untreated controls; quantification of (**e**) ATP and (**f**) HMGB1 release in cell culture supernatants after plasma treatment. Data are representative of three independent experiments. Statistical analysis was performed using two-way Anova (*p < 0.05; **p < 0.01; ***p < 0.001).

In addition to cytotoxic effects, therapeutic agents' capacity to enhance tumor immunogenicity to overcome tumor immune evasion is gaining interest in oncological research. One approach is the induction of immunogenic cell death (ICD), a regulated form of cell death accompanied by the timely release and surface expression of distinct molecules enhancing a broad antitumor immune response^39–41^. To compare the ability of kINPen and ^V^jet plasma to elicit ICD in tumor cells, we investigated apoptotic cell death (Fig. 3a) and attendant upregulation of critical ICD markers. Plasma-treatment was confirmed to be pro-apoptotic as indicated by increased translocation of phosphatidylserine to the outer leaflet of the cell membrane (Fig. 3b), with the ^V^jet plasma being superior cytotoxic in accordance to our findings in 3D tumor spheroids. Relative quantification of ICD-related molecules CRT (Fig. 3c), HSP70, and 90 on the surface of kINPen plasma-treated tumor cells were inferior to ^V^jet treatment for shorter treatment times and vice versa (Fig. 3d). Analysis of soluble molecules in collected cell culture supernatants revealed a dose-dependent increase in tumor-cell derived chemoattractants ATP (Fig. 3e) and HMGB1 (Fig. 3f). Again, 60 s exposure to kINPen plasma indicated a superior efficacy compared to equivalent ^V^jet treatment times.

### Tumor cell immunogenicity and reduction of tumor growth in vivo in the TUM-CAM assay following plasma treatment

**Figure 4:**
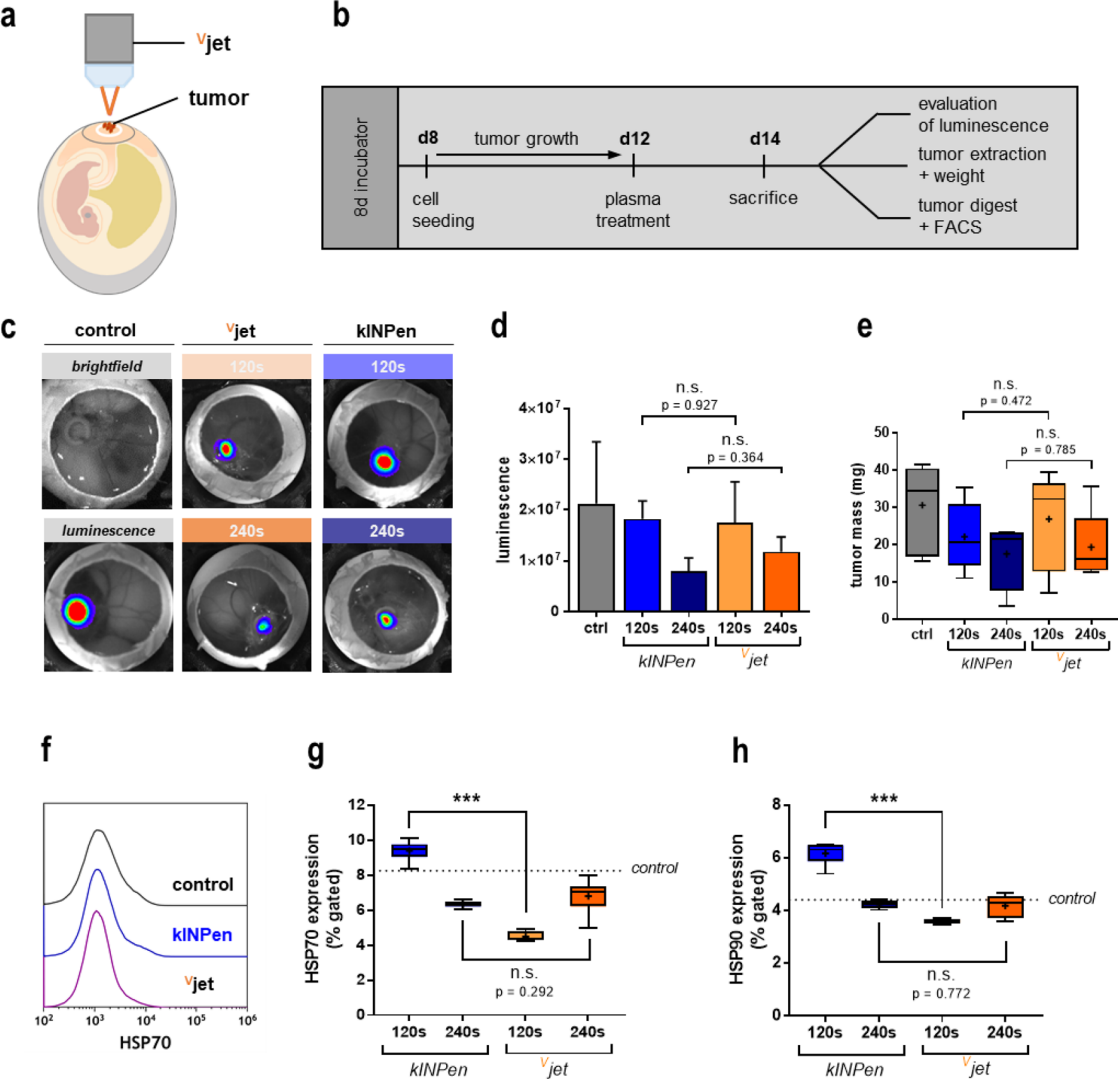
Tumor cell immunogenicity and reduction of tumor growth in vivo in the TUM-CAM assay following plasma treatment. (**a**) Schematic overview of plasma treatment of tumors grown on the CAM (**b**) and experimental procedure of the TUM-CAM model; (**c**–**d**) representative images of in vivo bioluminescent imaging of CT26 tumors 48 h after plasma treatment (**c**) and quantification thereof (**d**); (**e**) tumor mass of explanted in vivo tumors; (**f**) representative flow cytometry intensity histogram of HSP70 expression; (**g**) quantification of HSP70, and (**h**) HSP90 expression on tumor live cells digested from tumors of the TUM-CAM experiment. Boxplots show minimum to maximum with the mean indicated as (+). Data are from 5–7 eggs per group. Statistical analysis was performed using t-test (***p < 0.001). n.s. = non-significant.

**Figure 5:**
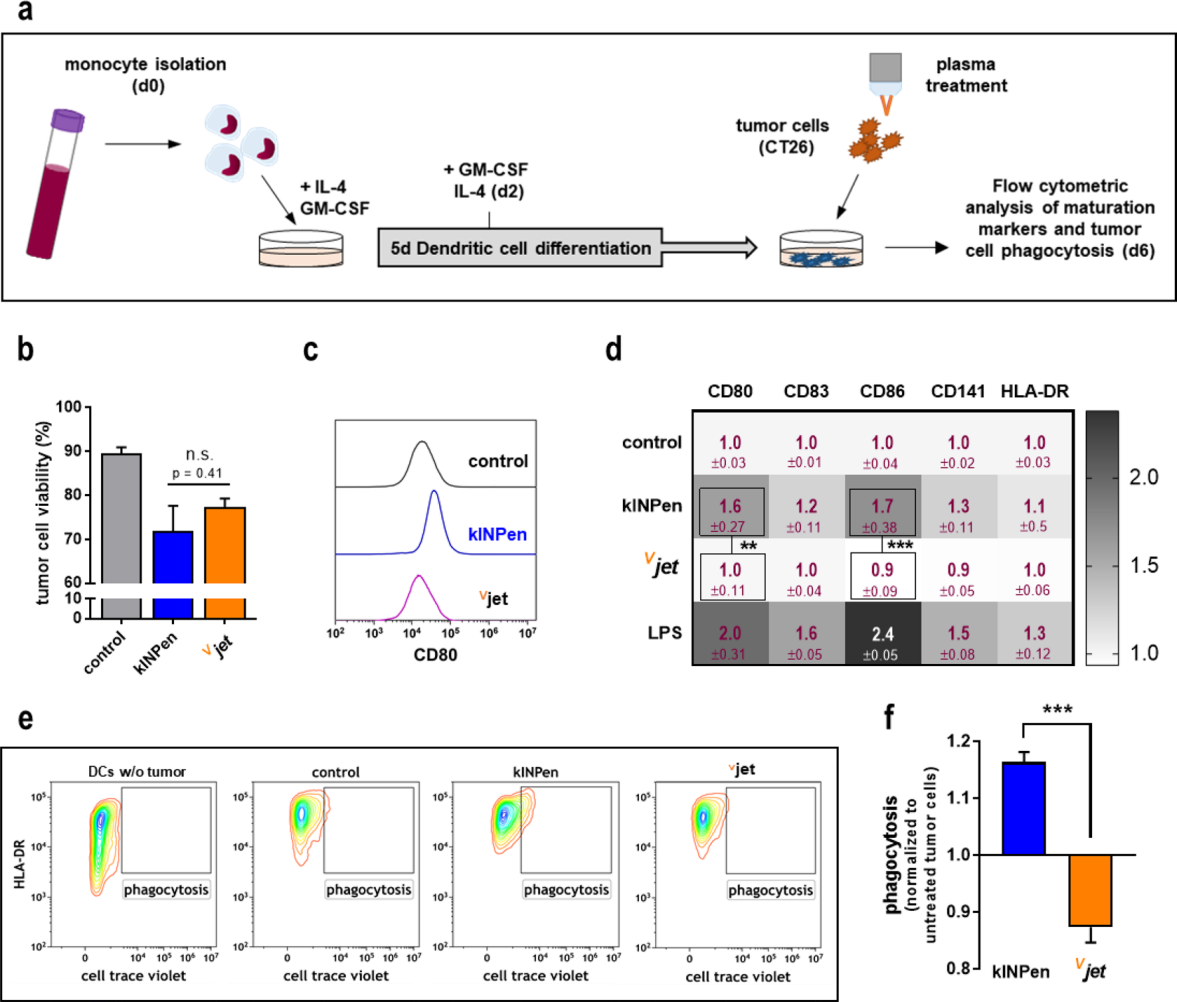
Tumor cell co-culture with and uptake by dendritic cells following plasma treatment. (**a**) Schematic overview of experimental procedure of co-culture experiments; (**b**) tumor cell viability 24 h after plasma treatment in co-culture with dendritic cells (DCs); (**c**) representative flow cytometry intensity histogram of CD80 expression on DCs after co-culture with plasma-treated tumor cells (CT26), and (**d**) quantification of maturation markers; Heatmap shows fold change of MFI normalized to control. (**e**–**f**) Representative flow cytometry contour plots of DC tumor uptake (e, and quantification (**f**). Data are representative of three independent experiments. Statistical analysis was performed using two-way anova or t-test (**p < 0.01; ***p < 0.001); n.s. = non-significant; LPS = lipopolysaccharide.

We used the tumor-chorion-allantois membrane (TUM-CAM) model to validate our findings in a semi in vivo model (Fig. 4a). Due to the rich vascularization of fertilized chicken eggs and their immunodeficiency, the TUM-CAM model is suited to investigate tumor therapies in living systems. CT26 cells were seeded on the CAM, allowed to grow, exposed to the plasma, and investigated 48 h later (Fig. 4b). In vivo bioluminescence imaging (Fig. 4c) indicated decreased tumor volumes of plasma-treated tumors compared to control tumors without significant difference between both treatment regimens (Fig. 4d). After tumor excision, we confirmed those findings by examining tumor weight (Fig. 4e). To investigate whether plasma treatment induced immunogenic cell death (ICD) in vivo, solid tumors were digested immediately after excision to generate single-cell suspensions. Flow cytometry was used to investigate heat-shock proteins' surface expression (HSP) as markers of immunogenicity (Fig. 4f) in the suspensions' live-cell fraction. HSP70 (Fig. 4g) and HSP90 (Fig. 4h) surface expression were exclusively increased on tumor cells after 120 s kINPen plasma treatment, while both 240 s of kINPen plasma treatment, as well as 120 s and 240 s of ^V^jet plasma treatment, did not potentiate HSP expression on the colorectal cancer cells. The main trait of ICD is increased phagocytosis by and activation of professional antigen-presenting cells (APCs), such as dendritic cells (DCs), with subsequent induction of maturation^6^. Hence, we co-cultured untreated or plasma-treated tumor cells with DCs (Fig. 5a) using synchronized treatment times that yielded similar cytotoxicity (Fig. 5b). Next, flow cytometry was used to investigate activation-related surface molecule expression on DCs co-cultured with the tumor cells (Fig. 5c). The expression of the maturation markers CD80, CD83, CD86, CD141, and HLA-DR was quantitative compared (Fig. 5d). Each marker's surface expression was normalized to their respective expression on DCs co-cultured with untreated tumor cells. Interestingly, challenging DCs with kINPen plasma-treated tumor cells resulted in the subsequent activation of the former. This was confirmed by treatment of DCs with lipopolysaccharides (LPS) as a positive control. By contrast, tumor cells exposed to the ^V^jet plasma were not able to induce DC maturation. To evaluate the capacity of DCs to perform phagocytosis, tumor cells were stained with cell trace violet (CTV) before co-culture to monitor tumor cell uptake. HLA-DR^+^/CTV^+^ cells were gated as DCs that had incorporated tumor material (Fig. 5e). The kINPen plasma-treated tumor cells showed increased uptake rates into DCs, whereas a decline in uptake was observed for the ^V^jet plasma-treated tumor cells (Fig. 5f). In sum, our findings indicate that exposure to kINPen plasma was superior to the ^V^jet plasma in the induction of ICD.

### Assessment of the irritation potential using hyperspectral imaging of the CAM

**Figure 6:**
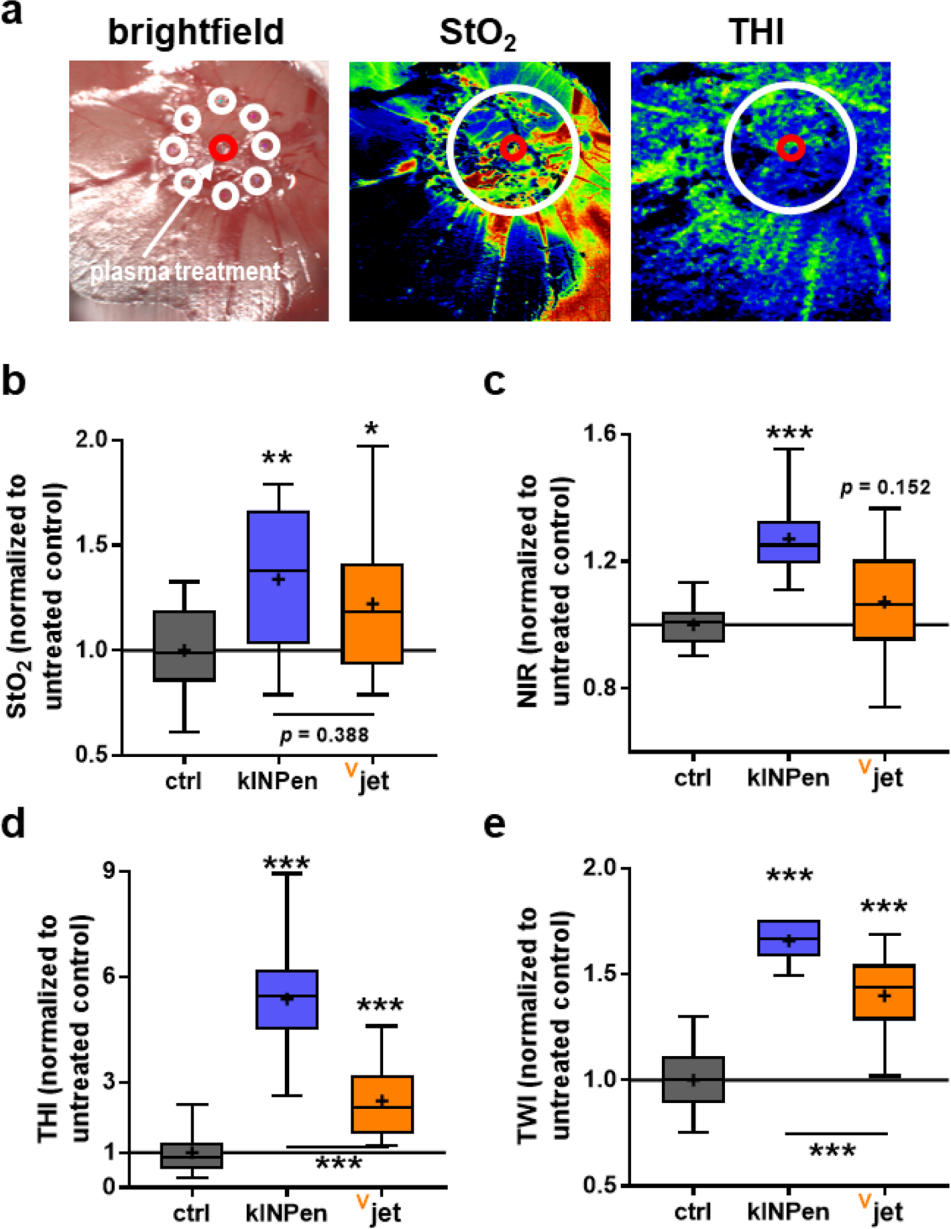
Assessment of the irritation potential using hyperspectral imaging of the CAM. (**a**) representative bright field and false-colored RGB images of kINPen treated CAM, and the red circle represents the plasma treatment site while the white circles represent the region in which eight ROIs were placed for quantification; (**b**–**e**) spectral data analysis to evaluate superficial tissue oxygenation (StO_2_, **b**), near-infrared perfusion index (NIR, **c**), tissue hemoglobin index (THI, **d**), and tissue water index (TWI, **e**). Spectral absorbance was normalized to untreated CAM. Data are min–max boxplots from 5 eggs per group. Statistical analysis was performed using one-way anova (***p < 0.001).

Plasma jet applications not only need to be effective but also safe. To this end, we plasma-treated the CAM of chicken embryos to analyze changes in this membrane that are observed upon contact with irritating or toxic agents^42^. Hyperspectral imaging technology was used to assess the irritation, and several regions of interest were quantified across distinct spectral ranges (Fig. 6a). Superficial tissue oxygenation (StO_2_, Fig. 6b) was enhanced with both jets, while near-infrared perfusion index (NIR, Fig. 6c) with the ^V^jet differed non-significantly from untreated controls and significantly from kINPen treatments. For the tissue hemoglobin index (THI, Fig. 6d) and tissue water index (TWI, Fig. 6e), treatment with both jets caused an increase but for the ^V^jet to a significantly smaller extent compared to the kINPen. These data suggested that the plasma-treated CAM's overall changes and irritation were less pronounced with the ^V^jet, suggesting favorable overall tolerability than the kINPen treatment.

### Assessment of genotoxic safety by micronucleus assay using high throughput image cytometry

**Figure 7:**
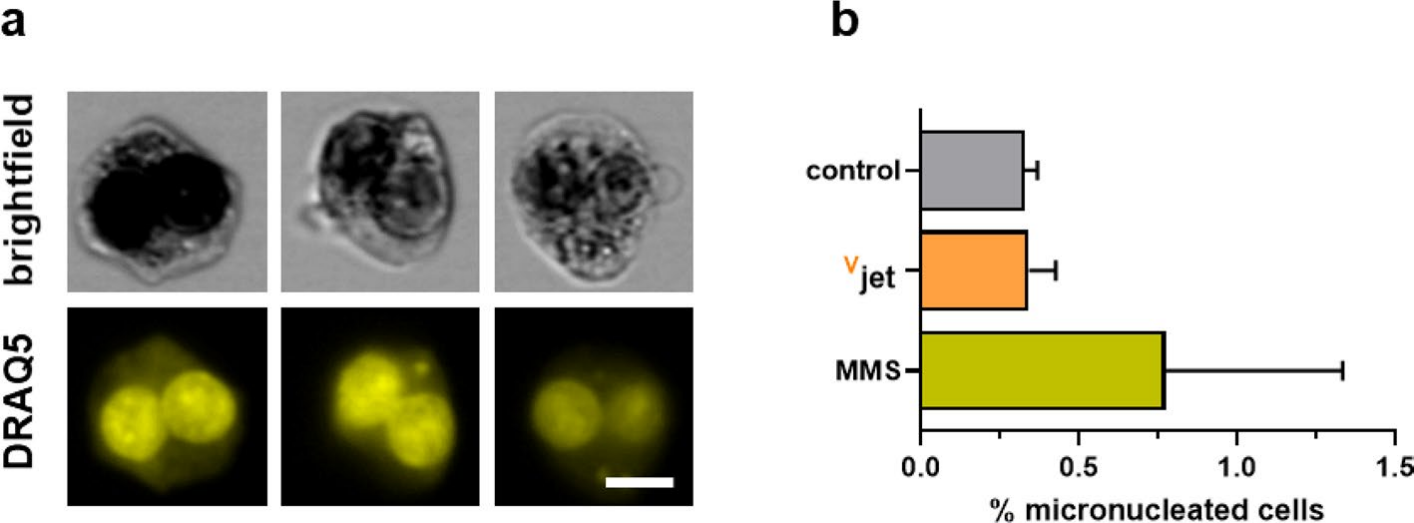
Assessment of genotoxic safety using an image flow cytometry-based micronucleus assay. (**a**) representative brightfield (BF) and DRAQ5 fluorescence images of binucleated cells having none (left), one (middle), and two (right) micronuclei in their cytosol, and (**b**) quantification of single micronucleated among all binucleated cells in the different conditions. Data are of three independent experiments.

To investigate the genotoxic safety of ^V^jet plasma treatment, we performed an image flow cytometry-based micronucleus assay, which is accredited by the Organization for Economic Development (OECD) [OECD, 1983] (Fig. 7a). Double-strand breaks (DSB) that occur in genetically injured cells can lead to micronuclei formation during the anaphase of the cell cycle that can be detected by nuclear staining in senescent cells. We used methyl methane sulfate (MMS), an alkylating agent inducing DNA-double strand breaks, as a positive control. Upon ^V^jet plasma treatment, we did not observe a significant increase in micronucleated cells, suggesting the ^v^jet plasma device to be void of genotoxic effects in vitro (Fig. 7b).

## Discussion

The field of plasma medicine continues to gain momentum in the biomedical field. While wound treatment with plasmas becomes an established routine across many dermatological centers in middle Europe, current research efforts investigate this technology's potential for other clinical applications, including cancer. We here present a novel neon-driven dual plasma jet for its potential in plasma onco-therapy. This was done by investigating this device's efficacy, mutagenicity, and irritation potential and side-by-side comparison across different biomedical assays with the benchmark plasma jet kINPen.

Both ^V^jet and kINPen were operated at 1 slm of neon and argon, respectively. Each assay was done with both jets tested in parallel to allow comparison. OES revealed the characteristic neon lines at 600 nm and above^43^, while the argon lines dominated above 700 nm^44^. In the UV range, the peaks of hydroxyl radicals at 307 nm^45^ and the second positive nitrogen system were relatively higher with the ^V^jet. As these data were normalized against the maximum peaks and since OES only is a semi-quantitative method, we next analyzed plasma-treated ddH_2_0 to compare the jets' reactive species generation quantitatively. Correlating with OES as found before for the kINPen^46^, the ^V^jet generated higher amounts of H_2_O_2_ and nitrogen species but not HOCl, species thought to be secondary rather than primary reaction products, although absolute differences were relatively modest. Consequently, it was not surprising to see overall comparable tumor inhibition rates in vivo and in 3D spheroids, with the ^V^jet being slightly more effective. The idea that the feed gas composition is tutorial in optimizing plasma effects in biomedical applications ranges back to the early 2000s^47^ but seemed not critical in our setup. This contrasts with other admixtures that were found to change the species output and cytotoxic consequences of plasma systems more dramatically in earlier studies of others and us^48–51^.

Plasma-induced cytotoxicity is thought to be rather pro-apoptotic^18,49,52,53^ as supported by increased activation of effector caspases in our study. Reactive species generated in the gas plasma phase can induce endoplasmic reticulum (ER) stress resulting in increased calcium influx into the mitochondria and mitochondria-dependent apoptosis^53,54^. Upon plasma-mediated activation of pro-apoptotic signaling cascades, an increase of Bax over Bcl2^55^, the release of mitochondrial cytochrome c^56^, and subsequent activation of p53 have been observed^57^. Alterations in signaling pathways in response to oxidative stress^58–60^ may impair plasma treatment efficacy to display cytotoxicity in different cancer cell lines. Besides tumor toxicity, immunological aspects become increasingly important in cancer therapy^61,62^. Similar to other technology-driven therapeutics such as photodynamic therapy^63^, the role of plasma treatment as an adjuvant treatment to stimulate immunity was also previously proposed^64–68^ and shown experimentally^24,67,69–71^. This is needed as many types of tumors develop molecular strategies to evade immune-mediated attacks^72^. The induction of immunogenic cell death (ICD) can elicit antitumor immune responses^6^.

In this study, we observed increased surface expression of critical ICD-related markers such as CRT, HSP70, and HSP90 in vitro and in vivo^8,73^*.* Analysis of soluble analytes in cell culture supernatants furthermore revealed the release of ATP and HMGB1, two strong chemoattractants for various immune cells^11,74,75^, in response to plasma treatment. However, ^V^jet plasma treatment was inferior in inducing ICD-related molecules HSP70 and HSP90 in in vivo-treated tumors. Interestingly, also extended treatment with kINPen plasma attenuated its efficacy in eliciting ICD in vivo. While cell death is required to initiate the release und ectopic expression of DAMPs, their translocation to the cell membrane's outer leaflet implies an unavoidable delay. Upon exposure to a maximal cytotoxic dose, cancer cells might dye to allow appropriate DAMP release quickly. Earlier investigations on hypericin-based photodynamic therapy's efficiency to elicit ICD indicated that a slower cell death maximizes the amount of generated DAMPs, while their release was diminished by rapid cell killing^76^. The observation that ICD might not be linearly dose-dependent and thus would require a submaximal treatment dose could be beneficial, as lower therapeutic doses would also secure healthy tissues from damage. However, it has to be investigated if milder treatment procedures can achieve the same therapeutic efficacy.

As the investigated molecules act as a chemoattractant to subsequently induce monocyte and DC maturation^77–79^, we thought to assess the immune stimulatory potential of plasma treatment in a co-culture assay of human peripheral blood-derived DCs and murine CT26 cells. The relevance of this cross-species approach is underlined by DC activation being triggered via the binding of immune-stimulatory molecules to evolutionarily conserved pattern recognition receptors such as toll-like receptors that only differ slightly in structure and subsequent signaling pathways across species^80,81^. Effects of plasma-derived species on myeloid cells such as DCs^82–84^, macrophages^85,86^ as well as innate lymphocytes^87^ was suggested before already.To gain evidence of the tumor treatment regimes' ability to induce DC maturation, we characterized the expression of reliable maturation markers on DCs. We found kINPen but not ^V^jet plasma-treated tumor cells to promote dendritic cell maturation and tumor cell phagocytosis. This is interesting, as cytotoxicity and reactive species generation were in a similar range. Possibly, other reactive species not investigated in our study contributed to this effect. For instance, a formation of peroxynitrite due to membrane-based oxidases was previously suggested in the plasma-treated liquid surrounding cells^88,89^, which could have taken place at different rate constants for both jets. Although a vast amount of ROS and RNS are produced in the plasma gas phase, as evident in the optical emission spectra of the respective devices, the influence of plasma-derived products in vitro depends majorly on a few long-lived molecules^37^. Those species represent the end-chemistry of the initially generated highly reactive species in the plasma-liquid-interphase. It is suggested that in vitro plasma effects are mainly mediated through H_2_O_2_^48^. Additionally, decomposition of RNS in the plasma-liquid-phase yields nitrite and nitrate generation that, despite their low reactivity, might act in synergy with H_2_O_2_^89^. The reaction of nitrite with H_2_O_2_ may lead to cytotoxic peroxynitrite production, which was not investigated in our study and might explain the differences observed with both devices despite their related liquid redox chemistry. However, the transfer of knowledge concerning the impact of selected species investigated in vitro is limited in vivo. As the plasma-liquid chemistry is of less importance, also short-lived species such as superoxide and hydroxyl radicals, that would rapidly deteriorate under in vitro culture conditions before reaching the cells, have to be taken into account^46^. Other parameters, such as electric fields, investigated as a mechanism contributing to plasma medicine effects^90–92^, might also be considered to explain the differences.

Oncological therapies not only need to be effective but also safe. The investigations on the ^V^jet performed are in agreement with the German DIN (German Institute of Standardization) specification 91315 named 'General requirements for medical plasma sources', which was published in 2014^93^. This developable specification describes obligatory and essential criteria for characterizing different biocompatible cold physical plasma devices operating at atmospheric pressure and intended for biomedical applications. As we have previously proposed^94^, this includes measurements of the temperature, patient leakage current, optical emission spectroscopy, and biology efficacy depending on the application. Additionally, we investigated the irritation potential on the CAM, a standard assay used in the pharmaceutical industry^95^, to estimate the inflammatory response of a living organism's sensitive surface membrane. This assay system was previously used with the kINPen 09^96^ but not in combination with hyperspectral imaging. This novel technology quantifies the superficial and deeper tissue oxygenation related to the hemoglobin content and the water content in tissues by mathematical modeling of spectral band intensities. Its technical details and successful use was demonstrated in previous studies in mice, volunteers, and patients^97–101^. Our data point to a significant increase in all parameters with both jets compared to the controls, while several parameters (NIR, THI, TWI) showed markedly elevated levels for the kINPen compared to the ^V^jet. This suggests the treatment with either of both plasma jets promoting an inflammatory response and increased vascularization or blood flow along with edema. For evaluation of mutagenicity, we performed the well-established micronucleus assay accredited by the Organization for Economic Development (OECD) [OECD, 1983]. Upon ^V^jet treatment, no signs of heavy chromosomal aberrations could be observed, indicative of a lack of genotoxicity.

Our data collectively demonstrate the ^V^jet to have potent antitumor effects that come with tolerable irritation of tissue. Compared to the kINPen, however, the ^V^jet showed reduced immunomodulatory potential. Moreover, in terms of the reactive species chemistry and concentrations deposited into liquids, both jets were within the same orders of magnitude, implying overall similar reaction pathways despite the different feed gases utilized.

## Materials and Methods

### Plasma sources

Plasma treatment was performed using the plasma sources kINPen (neoplas, Germany) and a newly designed ^V^jet. The kINPen plasma was generated using one standard liter per minute (slm) of argon (99.999% purity; Air liquid, France) as feed gas that was excited at the electrode at 1 MHz without duty cycle and generating power of 1–3 W^29^. The technical and chemical properties have been extensively described recently^30^. The reduced gas flow rate was necessary to reduce the mechanical impact on the biological test routines, especially the semi-in-vivo TUM-CAM model. For the measurements, a treatment distance of 8 mm was realized. The newly investigated device ^V^jet was operated with a gas flow of 1 slm neon (99.999% purity; Air liquid, France) distributed between two ceramic capillaries (Fig. S1a) and a total effluent length of 8 mm. The angle between the capillaries can be adjusted, while each capillary has a ring electrode wrapped around and is powered by each one side of a counter cycle resonator at a frequency 200 kHz and peak voltages up to 5°kV. The treatment distance was kept constant at 8 mm to operate within the cold plasma mode with temperatures at 40 °C (Fig. S1b) and negligible leakage current (Fig. S1c). The kINPen was also used at this distance showing a slightly higher temperature of 44 °C at 1 slm of argon and negligible leakage current.

### Plasma characterization

To compare both devices and control application relevant conditions, a selection of essential characterization was applied, comprising temperature measurement, patient leakage current measurement, and optical emission spectroscopy concerning DIN SPEC 91315^94^. For temperature measurement, an improved setup was applied^102^. A conductive copper surface is grounded via the patient leakage circuit from DIN EN 60601-1 via the commercial device UNIMET 800ST (Bender, Germany). In combination with the copper surface, a fiber optic temperature sensor (FOT Lab Kit; LumaSense Technologies, USA) was housed inside a copper tube attached to the surface to mediate the temperature warm up in case of plasma filaments interacting with a surface. 100 acquisitions for temperature and patient leakage current are performed via a homemade python based pc user interface and the mean, min, and max values were stored. Optical emission spectroscopy was performed for the investigation of plasma-derived products in the plasma-gas zone. Each of the plasma jets was positioned in front of a UV-sensitive optical emission spectrometer (AvaSpec-2048-USB2; Avantes, Germany) with a spectral resolution of 0.7 nm and end-on the plasma jet in the middle of the jet nozzle.

### Plasma-derived reactive species

To quantify ROS/RNS production in liquids, 4 ml of double-distilled water (ddH_2_O) were exposed to the effluent of either of the jets for 1 min, 5 min, and 10 min in a 6-well plate (Sarstedt, Germany). The evaporated volume was replaced with a predetermined amount of ddH_2_O to maintain iso-osmolarity. Immediately after plasma treatment, the analyses of the liquid was performed. Changes in the pH were measured using a pH-meter (Mettler-Toledo, Germany). For analysis of the hydrogen peroxide (H_2_O_2_) production in liquids, the *Amplex Ultra Red* assay (Thermo Scientific, Germany) was used according to the manufacturer's instruction. Fluorescence was determined using a multimode plate reader (F200; Tecan, Switzerland) at λ_ex_ 530 nm and λ_em_ 590 nm. The concentration of nitrite (NO_2_^-^) and nitrate (NO_3_^-^) was determined with the *Griess* assay (Cayman Chemical, Germany), and the absorbance was measured at 540 nm using a multiplate reader (M200; Tecan, Switzerland). Production of hypochlorous acid (HOCl) was measured using the taurine chloramine assay described before^67^. Briefly, samples were mixed with equal volumes of taurine (Sigma, Germany) buffer. A developer solution consisting of sodium acetate, sodium iodide, and tetramethylbenzidine in dimethylformamide was added immediately. Absorbance was measured at 645 nm. The kINPen operated with a helium/oxygen (He/O_2_) gas mixture was used as a positive control for HOCl production^67^. Absolute concentrations were calculated against a standard curve in all assays.

### Cell culture

Luciferase-expressing CT26 murine colon carcinoma cells (ATCC CRL-2638) were used in this study. Cells were maintained in Roswell Park Memorial Institute (RPMI) 1640 Medium (Pan Biotech, Germany) containing 10% fetal bovine serum, 1% glutamine, and 1% penicillin–streptomycin (all Sigma, Germany). Sub-culturing was performed every 2–3 days. Cells were kept at 37 °C, 95% humidity, and 5% CO_2_ in a cell culture incubator (Binder, Germany).

### Cellular viability and immunogenic cell death

To investigate cell death and concomitant upregulation of immunogenic surface markers, 1 × 10^5^ cells were seeded in 24-well flat-bottom plates and exposed to kINPen and ^V^jet plasma for 30 s and 60 s. Cells were detached with accutase 24 h after plasma treatment and stained with monoclonal antibodies (conjugate) targeted against calreticulin (Alexa Fluor 647), heat shock protein (HSP) 70 (Alexa Fluor 488), and HSP 90 (phycoerythrin; PE). Additionally, cells were stained with Annexin V (peridinin-chlorophyll-protein-cyanine; PerCp-Cy5.5) for detection of apoptotic cells. After washing, flow cytometric analysis was performed (CytoFLEX S; Beckman-Coulter, USA).

### Supernatant analysis

Analysis of soluble analytes in cell culture supernatants was carried out 1 h (ATP) or 24 h (HMGB1) after plasma treatment. For quantification of HMGB1, an enzyme-linked immune absorbance assay (ELISA; Hölzel, Austria) was carried out according to the manufacturer's protocol. To investigate ATP release in supernatants, an ATP Detection Assay Kit (Abcam, UK) was used following the supplier's instructions.

### 3D tumor spheroids

For spheroid formation, 3 × 10^3^ cells per well were seeded in a 96-well ultra-low-attachment spheroid formation plate (Thermo Fisher, Germany). After centrifugation, cells were cultured for three days. Before plasma treatment, spheroids were stained with Mitotracker Orange (MTO, final concentration 1 µM) and Sytox Green (SG, final concentration 1 µM; both Thermo Fisher, Germany) to quantify mitochondrial oxidation and terminally dead cells, respectively. Cells were then exposed to plasma for 15 s, 30 s, 60 s, and 90 s. Subsequently, imaging was performed at 0 h, 12 h, 24 h, 48 h, and 72 h using a high content imaging device (Operetta CLS; PerkinElmer, Germany) equipped with laser-based autofocus. Images were acquired in bright field and fluorescence channels (λ_ex_ 550 nm and λ_em_ 580 nm for MTO; λ_ex_ 490 nm and λ_em_ 520 nm for SG) using a 5 × air objective (NA = 0.16) and ten z-stacks per well and spheroid. Acquisition parameters were standardized in preliminary experiments to optimize image focus and signal-to-noise ratio. For image analysis, the bright field channel was inverted, and an intensity cut-off was defined to distinguish the spheroid area from the background. In that area, the mean fluorescence intensity (MFI) of MTO and SG was quantified. Experimental setup and image quantification were performed using the analysis software Harmony 4.9 (PerkinElmer, Germany; https://www.perkinelmer.com/).

### TUM-CAM model

The tumor chorion-allantois membrane model (TUM-CAM) was performed as previously described^103^. Ethical approval is not needed for this model if embryos are euthanized until day 15th of ontogenesis. Pathogen-free eggs (Valo BioMedia, Germany) were incubated for six days in a dedicated breeding incubator at 37 °C and 60% humidity. On day 6, the egg's pointed pole was carefully punctured using a cannula (20G) to create an air cell between the CAM and the eggshell. Two days later, eggs were carefully opened at the punctured side. 1 × 10^6^ cells in 15 µl matrigel were seeded in a silicone ring that was placed on the CAM. Eggs were incubated for another four days before plasma treatment for 120 s and 240 s with either of the jets. On day 14, tumor growth on the CAM of the living embryos was assessed in vivo using bioluminescent imaging (IVIS spectra S5; PerkinElmer) and ex vivo by weighing after tumor excision. According to the manufacturer's protocol, explored tumors were digested using the tumor dissociation enzyme mix and the OctaMACS technology (Miltenyi BioTec, Germany). Single-cell suspensions were stained with anti-mouse monoclonal antibodies (conjugate) targeting heat-shock protein (HSP) 70 (Alexa Fluor 594) and HSP90 (Alexa Fluor 700). After washing, flow cytometric analysis was performed (CytoFLEX S; Beckman-Coulter, Germany).

### Hyperspectral imaging

To analyze both plasma jets' irritation potential, the CAM was plasma-treated for 120 s, and several physiological parameters were recorded two days (2d) later using hyperspectral imaging camera system TIVITA Tissue (Diaspective Vision, Germany)^101^. This system can determine several tissue characteristics in real-time by mathematical modeling of series of bandpass wavelengths from 500–1000 nm without using any contrast agents. The software algorithms allow retrieving tissue oxygenation (StO_2_) in superficial layers, the perfusion into deeper tissue regions of 4–6 mm (near-infrared index, NIR), the tissue hemoglobin index (THI) with monitoring of the percental volume of hemoglobin of surface perfusion, and the tissue water index (TWI). The camera's working distance to the CAM was 50 cm, and each hyperspectral image was acquired under standardized conditions (darkroom and identical exposure times). Regions of interest (ROI) were defined to allow the camera-specific software TIVITA Suite 1.0.0.5 (Diaspective Vision, Germany; http://diaspective-vision.com/) to calculate parameters in well-defined circular areas. Normalization was done against the respective parameters of the untreated control.

### PBMC isolation and generation of monocyte-derived DCs

The local ethics committee at the University Medical Center approved this study (approval number: BB166/17). Human peripheral blood mononuclear cells (PBMCs) were isolated from buffy coats of healthy donors provided by the Institute of Transfusion Medicine Greifswald by density-gradient centrifugation using lymphocyte separation medium (VWR, Germany). Residual erythrocytes were lysed using a red blood cell (RBC) lysis buffer (Thermo Fisher, Germany). For monocyte isolation, CD14 microbeads (Miltenyi Biotec, Germany) were used according to the manufacturer's protocol. Flow cytometric (Attune NxT; Applied Biosystems, USA) verification of purity was performed immediately after isolation and was always greater than 85%. 1 × 10^5^ monocytes per well were seeded in 24-well plates (Sarstedt, Germany). The cell culture medium was supplemented with 800 IU granulocyte–macrophage colony-stimulating factor (GM-CSF) and 500 IU interleukin (IL) 4 (all PeproTech, Germany) to initiate monocyte-derived dendritic cells (DCs). After two days, cells were re-stimulated by adding the same amounts of GM-CSF and IL-4 to the cell culture medium. Immature DCs were used for co-culture experiments on day 5.

### DC maturation and phagocytosis

CT26 cells were stained with cell trace violet (final concentration 1 µM; Thermo Fisher, Germany) and seeded at 1 × 10^5^ cells per well in 24-well plates (Sarstedt, Germany) before exposure to the plasma of either of the jets for 30 s. Immediately after plasma treatment, tumor cells were collected and added to immature DCs. Twenty-four hours later, co-cultured cells were harvested and stained with monoclonal antibodies (conjugate) targeting human CD80 (phycoerythrin-dazzle), CD83 (PerCP-Cy5.5), CD86 (phycoerythrin), CD141 (brilliant violet 650), and HLA-DR (allophycocyanin-cy7) (all BioLegend, Netherlands). For live-dead discrimination at 808 nm excitation, iFluor maleimide (AAT Bioquest, USA) was added. After washing, flow cytometry was performed (CytoFLEX S; Beckman-Coulter, Germany), and cells were analyzed using Kaluza software 2.1.1 (Beckman-Coulter, Germany; https://www.beckman.de/). Tumor cells were identified by cell trace violet staining, while DCs were distinguished using HLA-DR expression. As a positive control, lipopolysaccharide (LPS, final concentration 100 ng/ml; Sigma, Germany) was used.

### Image flow cytometry micronucleus assay

The micronucleus assay was performed as described previously^31,104^. Briefly, 1 × 10^6^ TK6 cells per well were seeded in 24 well plates and exposed to ^v^jet plasma or control gas or the DNA-damaging agent methyl methane sulfate (MMS). Cytochalasin B (Sigma, Germany) was added 24 h later to block cytokinesis. After another 24 h of incubation, cells were detached, fixed with 4% paraformaldehyde, and lysed with 0.1% Triton X-100. After staining with DRAQ5, the relative frequencies of micronuclei in binucleated cells was assessed using imaging flow cytometry (ImageStream Mark II; Merck-Millipore, USA). Data analysis was performed using Ideas software 6.2 (Merck-Millipore, USA; https://www.luminexcorp.com/).

### Statistical analysis

Data are from three independent experiments with at least three technical replicates each. Graphing and statistical analysis were done using prism 8.4 (GraphPad Software, USA; https://www.graphpad.com/) and either *t*-test or one-way or two-way analysis of variances (anova) as indicated. Data show mean ± standard error of the mean (SEM) if not indicated otherwise in the figure legends. The levels of significance were indicated as follows: α = 0.05 (*), α = 0.01 (**), α = 0.001 (***).

## Supplementary Information

Supplementary Information.

## References

[1] Bray F (2018). Global cancer statistics 2018: GLOBOCAN estimates of incidence and mortality worldwide for 36 cancers in 185 countries. CA Cancer J. Clin..

[2] Trachootham D, Alexandre J, Huang P (2009). Targeting cancer cells by ROS-mediated mechanisms: a radical therapeutic approach?. Nat. Rev. Drug Disc..

[3] Sundaresan M (1995). Requirement for generation of H2O2 for platelet-derived growth factor signal transduction. Science.

[4] Bae YS (1997). Epidermal growth factor (EGF)-induced generation of hydrogen peroxide. Role in EGF receptor-mediated tyrosine phosphorylation. J. Biol. Chem..

[5] Denu JM, Tanner KG (1998). Specific and reversible inactivation of protein tyrosine phosphatases by hydrogen peroxide: evidence for a sulfenic acid intermediate and implications for redox regulation. Biochemistry.

[6] Kroemer G (2013). Immunogenic cell death in cancer therapy. Annu. Rev. Immunol..

[7] Kepp O (2014). Consensus guidelines for the detection of immunogenic cell death. Oncoimmunology.

[8] Fucikova J (2014). High hydrostatic pressure induces immunogenic cell death in human tumor cells. Int. J. Cancer.

[9] Basu S (2001). CD91 is a common receptor for heat shock proteins gp96, hsp90, hsp70, and calreticulin. Immunity.

[10] Galluzzi L (2020). Consensus guidelines for the definition, detection and interpretation of immunogenic cell death. J. Immunother. Cancer.

[11] Garg AD (2011). DAMPs and PDT-mediated photo-oxidative stress: exploring the unknown. Photochem. Photobiol. Sci..

[12] Krysko DV (2012). Immunogenic cell death and DAMPs in cancer therapy. Nat. Rev. Cancer.

[13] Yokoyama C (2017). Induction of oxidative stress by anticancer drugs in the presence and absence of cells. Oncol. Lett..

[14] Ramanathan B (2005). Resistance to paclitaxel is proportional to cellular total antioxidant capacity. Cancer Res..

[15] Hosokawa S (2018). Photodynamic therapy in patients with head and neck squamous cell carcinoma. Lasers Surg. Med..

[16] Simone NL (2009). Ionizing radiation-induced oxidative stress alters miRNA expression. PLoS ONE.

[17] Adamovich I (2017). The 2017 Plasma Roadmap: Low temperature plasma science and technology. J. Phys. D-Appl. Phys..

[18] Liedtke KR (2018). Cold physical plasma selectively elicits apoptosis in murine pancreatic cancer cells in vitro and in ovo. Anticancer Res.

[19] Kaushik N (2015). Responses of solid tumor cells in DMEM to reactive oxygen species generated by non-thermal plasma and chemically induced ROS systems. Sci. Rep..

[20] Ishaq M, Evans MM, Ostrikov KK (2014). Effect of atmospheric gas plasmas on cancer cell signaling. Int. J. Cancer.

[21] Vermeylen S (2016). Cold atmospheric plasma treatment of melanoma and glioblastoma cancer cells. Plasma Process. Polymers.

[22] Schneider C (2018). Cold atmospheric plasma treatment inhibits growth in colorectal cancer cells. Biol. Chem..

[23] Freund E (2019). Physical plasma-treated saline promotes an immunogenic phenotype in CT26 colon cancer cells in vitro and in vivo. Sci. Rep..

[24] Lin AG (2018). Non-thermal plasma induces immunogenic cell death in vivo in murine CT26 colorectal tumors. Oncoimmunology.

[25] Pasqual-Melo G (2020). Plasma treatment limits cutaneous squamous cell carcinoma development in vitro and in vivo. Cancers (Basel).

[26] Metelmann H-R (2018). Clinical experience with cold plasma in the treatment of locally advanced head and neck cancer. Clin. Plasma Med..

[27] Schuster M (2016). Visible tumor surface response to physical plasma and apoptotic cell kill in head and neck cancer. J. Craniomaxillofac. Surg..

[28] Metelmann HR (2018). Treating cancer with cold physical plasma: On the way to evidence-based medicine. Contrib. Plasma Phys..

[29] Bekeschus S (2016). The plasma jet kINPen – A powerful tool for wound healing. Clin. Plasma Med..

[30] Reuter S, von Woedtke T, Weltmann KD (2018). The kINPen-a review on physics and chemistry of the atmospheric pressure plasma jet and its applications. J. Phys. D-Appl. Phys..

[31] Bekeschus S (2018). High throughput image cytometry micronucleus assay to investigate the presence or absence of mutagenic effects of cold physical plasma. Environ. Mol. Mutagen.

[32] Wende K (2016). Risk assessment of a cold argon plasma jet in respect to its mutagenicity. Mutat. Res. Genet. Toxicol. Environ. Mutagen.

[33] Bekeschus S (2019). Risk assessment of kINPen plasma treatment of four human pancreatic cancer cell lines with respect to metastasis. Cancers (Basel).

[34] Privat-Maldonado A (2019). ROS from physical plasmas: redox chemistry for biomedical therapy. Oxid. Med. Cell Longev.

[35] von Woedtke T (2019). Plasma medicine: a field of applied redox biology. Vivo.

[36] Hasse S (2020). Plasma treatment limits human melanoma spheroid growth and metastasis independent of the ambient gas composition. Cancers (Basel).

[37] Bruggeman PJ (2016). Plasma–liquid interactions: a review and roadmap. Plasma Sources Sci. Technol..

[38] Winter J (2013). Feed gas humidity: a vital parameter affecting a cold atmospheric-pressure plasma jet and plasma-treated human skin cells. J. Phys. D-Appl. Phys..

[39] Obeid M (2007). Ecto-calreticulin in immunogenic chemotherapy. Immunol. Rev..

[40] Obeid M (2007). Calreticulin exposure dictates the immunogenicity of cancer cell death. Nat. Med..

[41] Fucikova J (2018). Relevance of the chaperone-like protein calreticulin for the biological behavior and clinical outcome of cancer. Immunol. Lett..

[42] Luepke NP (1985). Hen's egg chorioallantoic membrane test for irritation potential. Food Chem. Toxicol..

[43] Yamada H (2020). Striation phenomena in a low temperature atmospheric pressure neon plasma jet by optical emission spectroscopy. Phys. Plasmas.

[44] Wiegand C (2014). Antimicrobial impact of cold atmospheric pressure plasma on medical critical yeasts and bacteria cultures. Skin Pharmacol. Physiol..

[45] Hong YJ (2012). Measurement of hydroxyl radical density generated from the atmospheric pressure bioplasma jet. J. Instrum..

[46] Bekeschus S (2017). Basic research in plasma medicine—a throughput approach from liquids to cells. J. Vis. Exp..

[47] Purevdorj D (2003). Effect of feed gas composition of gas discharge plasmas on *Bacillus pumilus* spore mortality. Lett. Appl. Microbiol..

[48] Wende K (2015). Identification of the biologically active liquid chemistry induced by a nonthermal atmospheric pressure plasma jet. Biointerphases.

[49] Bekeschus S (2017). Oxygen atoms are critical in rendering THP-1 leukaemia cells susceptible to cold physical plasma-induced apoptosis. Sci. Rep..

[50] Hertwig C (2015). Decontamination of whole black pepper using different cold atmospheric pressure plasma applications. Food Control.

[51] Deng S (2008). Bacterial inactivation by atmospheric pressure dielectric barrier discharge plasma jet. Jpn. J. Appl. Phys..

[52] Bekeschus S (2020). Ex vivo exposure of human melanoma tissue to cold physical plasma elicits apoptosis and modulates inflammation. Appl. Sci.-Basel.

[53] Ahn HJ (2011). Atmospheric-pressure plasma jet induces apoptosis involving mitochondria via generation of free radicals. PLoS ONE.

[54] Ruwan Kumara MH (2016). Non-thermal gas plasma-induced endoplasmic reticulum stress mediates apoptosis in human colon cancer cells. Oncol. Rep..

[55] Turrini E (2017). Cold Atmospheric plasma induces apoptosis and oxidative stress pathway regulation in T-lymphoblastoid leukemia cells. Oxid. Med. Cell Longev.

[56] Gandhirajan RK (2018). Cytochrome C oxidase inhibition and cold plasma-derived oxidants synergize in melanoma cell death induction. Sci. Rep..

[57] Hayashi N (2018). Activation of p53-mediated apoptosis pathway in HSC3 cancer cell irradiated by atmospheric DBD oxygen plasma. IEEE Trans. Plasma Sci..

[58] Smolkova B (2019). Non-thermal plasma, as a new physicochemical source, to induce redox imbalance and subsequent cell death in liver cancer cell lines. Cell Physiol. Biochem..

[59] Bekeschus S (2020). xCT (SLC7A11) expression confers intrinsic resistance to physical plasma treatment in tumor cells. Redox Biol..

[60] Bekeschus S (2018). Hmox1 upregulation is a mutual marker in human tumor cells exposed to physical plasma-derived oxidants. Antioxidants (Basel).

[61] Tay RE, Richardson EK, Toh HC (2020). Revisiting the role of CD4(+) T cells in cancer immunotherapy-new insights into old paradigms. Cancer Gene Ther..

[62] Galon J, Bruni D (2019). Approaches to treat immune hot, altered and cold tumours with combination immunotherapies. Nat. Rev. Drug Discov..

[63] Falk-Mahapatra R, Gollnick SO (2020). photodynamic therapy and immunity: an update. Photochem. Photobiol..

[64] Bekeschus S, Clemen R, Metelmann H-R (2018). Potentiating anti-tumor immunity with physical plasma. Clin. Plasma Med..

[65] Miller V, Lin A, Fridman A (2015). Why target immune cells for plasma treatment of cancer. Plasma Chem. Plasma Process..

[66] Witzke K (2020). Plasma medical oncology: immunological interpretation of head and neck squamous cell carcinoma. Plasma Process. Polymers.

[67] Bekeschus S (2020). Medical gas plasma jet technology targets murine melanoma in an immunogenic fashion. Adv. Sci. (Weinh.).

[68] Clemen R, Bekeschus S (2020). Oxidatively modified proteins: cause and control of diseases. Appl. Sci..

[69] Lin A (2019). Non-thermal plasma as a unique delivery system of short-lived reactive oxygen and nitrogen species for immunogenic cell death in melanoma cells. Adv. Sci. (Weinh.).

[70] Mizuno K (2018). Plasma-induced suppression of recurrent and reinoculated melanoma tumors in mice. IEEE TRPMS.

[71] Freund E, Bekeschus S (2020). Gas plasma-oxidized liquids for cancer treatment: pre-clinical relevance, immuno-oncology, and clinical obstacles. IEEE Trans. Radiat. Plasma Med. Sci..

[72] Saleh R, Elkord E (2019). Acquired resistance to cancer immunotherapy: Role of tumor-mediated immunosuppression. Semin. Cancer Biol..

[73] Bekeschus S (2019). Physical plasma-triggered ROS induces tumor cell death upon cleavage of HSP90 chaperone. Sci. Rep..

[74] Garg AD (2010). Immunogenic cell death, DAMPs and anticancer therapeutics: an emerging amalgamation. Biochim. Biophys. Acta.

[75] Garg AD (2012). A novel pathway combining calreticulin exposure and ATP secretion in immunogenic cancer cell death. EMBO J..

[76] Doix B (2019). Low photosensitizer dose and early radiotherapy enhance antitumor immune response of photodynamic therapy-based dendritic cell vaccination. Front. Oncol..

[77] Garg AD (2012). Hypericin-based photodynamic therapy induces surface exposure of damage-associated molecular patterns like HSP70 and calreticulin. Cancer Immunol. Immunother..

[78] Thériault JR (2005). Extracellular HSP70 binding to surface receptors present on antigen presenting cells and endothelial/epithelial cells. FEBS Lett..

[79] Calderwood SK (2007). Extracellular heat shock proteins in cell signaling. FEBS Lett..

[80] Zilionis R (2019). Single-cell transcriptomics of human and mouse lung cancers reveals conserved myeloid populations across individuals and species. Immunity.

[81] Medzhitov R (2001). Toll-like receptors and innate immunity. Nat. Rev. Immunol..

[82] Pasqual-Melo G (2020). Combination of Gas plasma and radiotherapy has immunostimulatory potential and additive toxicity in murine melanoma cells in vitro. Int. J. Mol. Sci..

[83] Bekeschus S (2020). Gas plasma-treated prostate cancer cells augment myeloid cell activity and cytotoxicity. Antioxidants (Basel).

[84] Freund E (2019). Plasma-derived reactive species shape a differentiation profile in human monocytes. Appl. Sci.-Basel.

[85] Bekeschus S (2017). Environmental control of an argon plasma effluent and its role in THP-1 monocyte function. IEEE Trans. Plasma Sci..

[86] Khabipov A (2019). RAW 264.7 macrophage polarization by pancreatic cancer cells—a model for studying tumour-promoting macrophages. Anticancer Res..

[87] Clemen R (2020). Physical plasma-treated skin cancer cells amplify tumor cytotoxicity of human natural killer (NK) cells. Cancers.

[88] Girard F (2016). Formation of reactive nitrogen species including peroxynitrite in physiological buffer exposed to cold atmospheric plasma. RSC Adv..

[89] Bauer G, Graves DB (2016). Mechanisms of selective antitumor action of cold atmospheric plasma-derived reactive oxygen and nitrogen species. Plasma Process. Polymers.

[90] Babaeva NY, Kushner MJ (2010). Intracellular electric fields produced by dielectric barrier discharge treatment of skin. J. Phys. D-Appl. Phys..

[91] Norberg SA, Johnsen E, Kushner MJ (2016). Helium atmospheric pressure plasma jets interacting with wet cells: delivery of electric fields. J. Phys. D: Appl. Phys..

[92] Simoncelli E (2019). Experimental investigation on the influence of target physical properties on an impinging plasma jet. Plasma.

[93] (91315), D.-S., *General Requirements for Medical Plasma Sources* (Beuth-Verlag, 2014).

[94] Mann MS (2016). Introduction to DIN-specification 91315 based on the characterization of the plasma jet kINPen® MED. Clin. Plasma Med..

[95] Steiling W (1999). The HET-CAM, a useful in vitro assay for assessing the eye irritation properties of cosmetic formulations and ingredients. Toxicol. In Vitro.

[96] Bender C (2010). The irritation potential of nonthermal atmospheric pressure plasma in the HET-CAM. Plasma Process. Polymers.

[97] Rutkowski R (2020). Long-term risk assessment for medical application of cold atmospheric pressure plasma. Diagnostics (Basel).

[98] Schmidt, A., *et al.* Hyperspectral Imaging of Wounds Reveals Augmented Tissue Oxygenation Following Cold Physical Plasma Treatment in Vivo. IEEE TRPMS, 1–1 (2020).

[99] Daeschlein, G., *et al.* Hyperspectral Imaging: Innovative Diagnostics to Visualize Hemodynamic Effects of Cold Plasma in Wound Therapy. Biomed Tech (Berlin, 2018). 10.1515/bmt-2017-0085 29727297

[100] Schmidt A (2020). The molecular and physiological consequences of cold plasma treatment in murine skin and its barrier function. Free Radic. Biol. Med..

[101] Kulcke, A., *et al.* A Compact Hyperspectral Camera for Measurement of Perfusion Parameters in Medicine. Biomed Tech (Berlin, 2018). 10.1515/bmt-2017-0145 29522415

[102] Timmermann E (2021). Piezoelectric-driven plasma pen with multiple nozzles used as a medical device: risk estimation and antimicrobial efficacy. J. Phys. D: Appl. Phys..

[103] Liedtke KR (2020). Gas plasma-conditioned ringer's lactate enhances the cytotoxic activity of cisplatin and gemcitabine in pancreatic cancer in vitro and in ovo. Cancers (Basel).

[104] Bekeschus S (2019). Elevated H2AX phosphorylation observed with kINPen plasma treatment is not caused by ROS-mediated DNA damage but is the consequence of apoptosis. Oxid. Med. Cell Longev.

