# Supplementary material for: Tumor cytotoxicity and immunogenicity of a novel V-jet neon plasma source compared to the kINPen
Source: Sci Rep. 2021 Jan 8;11:136. doi: 10.1038/s41598-020-80512-w (PMC7794240; doi:10.1038/s41598-020-80512-w)
Supplement: Supplementary file 1 — Supplementary Information. [file 41598_2020_80512_MOESM1_ESM.docx]

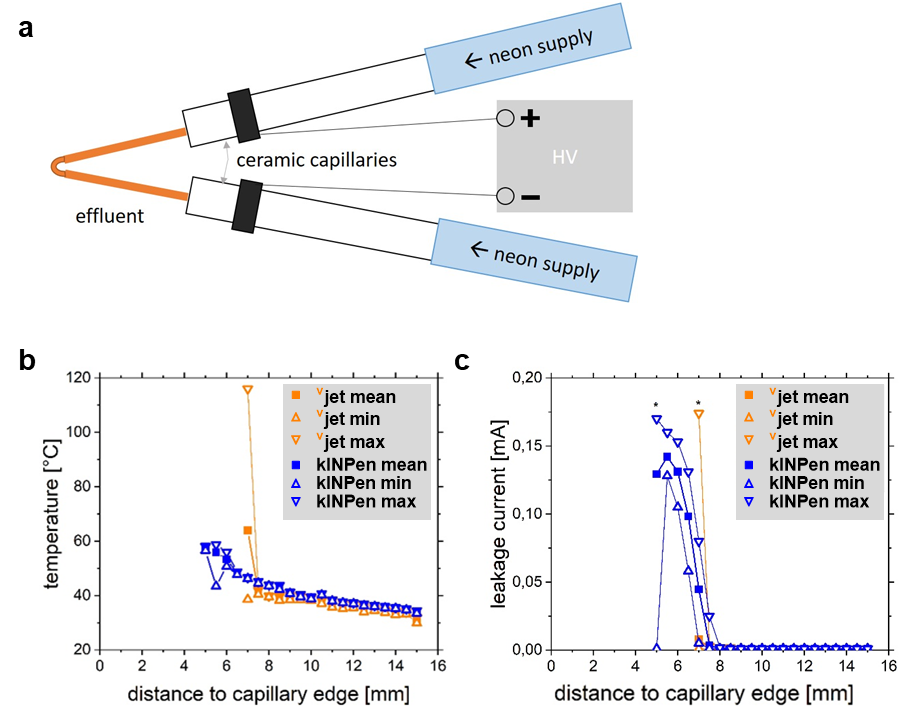


**Supplementary figure S1. Plasma device and basic operation parameters.** (a) scheme of the ^V^jet plasma device; (b) temperature value for each device under investigation for different distances, each showing a mean value of 100 measurements, the minimal and maximal measured value; (c) patient leakage current measurement for each device under investigation for different distances including the mean, the minimal and maximal measured value, * indicates measurement abortion due to devices reaching unstable conditions.
